# Positive Psychology and Disability Studies: Directions for Research on Aging With IDD

**DOI:** 10.1093/geroni/igab046.867

**Published:** 2021-12-17

**Authors:** Lieke van Heumen, Tamar Heller

**Affiliations:** 1 University of Illinois at Chicago, Chicago, Illinois, United States; 2 University of Illinois at chicago, Department of disability and Human Development, University of Illinois at Chicago, Illinois, United States

## Abstract

This presentation outlines future directions for research on aging with intellectual and developmental disabilities (IDD) informed by positive psychology and disability studies theory. Research on aging with IDD often focuses on losses in functioning that come with age and on ways to prevent such decline. When applying a positive psychology lens to the experience of aging with IDD the focus shifts to determinants of positive emotions and strength and on ways of increasing meaningfulness in life as one ages. A positive psychology lens also relies on the assumption that people can self-direct and organize their lives, which is essential for individuals aging with IDD who historically have lacked opportunities to exercise self-determination. Positive psychology as applied to disability aligns with disability studies perspectives that emphasize that different ways of being in the world can be ‘sources of knowledge, satisfaction, creativity, and happiness’ (Chivers, 2011 p.9).

